# A Clinical Scale for Rating the Severity of Bulbar Lower Motor Neuron Dysfunction in Amyotrophic Lateral Sclerosis

**DOI:** 10.3390/biomedicines11072039

**Published:** 2023-07-20

**Authors:** Stefano Zoccolella, Alessia Giugno, Giammarco Milella, Marco Filardi, Alessandro Introna, Angela Fraddosio, Eustachio D’Errico, Valentina Gnoni, Ludovica Tamburrino, Daniele Urso, Francesca Caputo, Salvatore Misceo, Giancarlo Logroscino

**Affiliations:** 1 Neurology Unit, San Paolo Hospital, Azienda Sanitaria Locale (ASL) Bari, 70132 Bari, Italy; salvatore.misceo@asl.bari.it; 2 Center for Neurodegenerative Diseases and the Aging Brain, University of Bari Aldo Moro at Pia Fondazione “Card. G. Panico”, 73039 Tricase, Italy; giugnoalessia89@gmail.com (A.G.); marco.filardi@uniba.it (M.F.); gnoni.vale@gmail.com (V.G.); ludo.tamburrino@gmail.com (L.T.); d.urso@piafondazionepanico.it (D.U.); giancarlo.logroscino@uniba.it (G.L.); 3 Department of Translational Biomedicine and Neurosciences (DiBraiN), University of Bari Aldo Moro, 70121 Bari, Italy; giammarco.milella91@gmail.com (G.M.); ale.introna@gmail.com (A.I.); anfraddo@libero.it (A.F.); eus1980@libero.it (E.D.); caputofrancesca90@gmail.com (F.C.)

**Keywords:** amyotrophic lateral sclerosis, lower motor neuron, bulbar region, survival

## Abstract

Background: Amyotrophic lateral sclerosis (ALS) is characterized by the progressive loss of upper (UMN) and lower motor neurons (LMN) in four different body regions (bulbar, cervical, thoracic, and lumbosacral). Over the past decades, several clinical scoring systems have been developed to assess the UMN and LMN burden in ALS. However, concerning the bulbar LMN burden, the available scoring systems solely assess the presence/absence of bulbar LMN signs without providing a degree of impairment. Therefore, in this study, we proposed a novel scale to stratify subjects with ALS according to the bulbar LMN involvement and assessed its prognostic value. Methods: We developed a four-item scale based on the LMN signs according to the El Escorial criteria. Ten raters, specializing in ALS or neurocognitive disorders, retrospectively applied the scale to the first evaluation of 195 patients with ALS. Cohen’s kappa (Cohen’s *k*) and an intra-class correlation coefficient (ICC) were used to assess the inter-rater reliability. The Kaplan–Mayer estimator was used to estimate survival distribution according to the bulbar scale scores. Results: The raters showed a substantial to excellent agreement with Cohen’s *k*, ranging from 0.834 to 0.975, with an overall ICC of 0.922 (95% CI = 0.906–0.936). The survival distribution was statistically different across the three bulbar scale scores (χ^2^_(2)_ = 9.50, *p* < 0.01). Conclusions: Our bulbar LMN scale represents a reliable measure of the bulbar LMN signs in ALS. This easy-to-administer clinical scale could provide unique information in phenotyping and predicting survival in ALS.

## 1. Introduction

Amyotrophic lateral sclerosis (ALS) is a heterogeneous neurodegenerative disease characterized by the progressive loss of motor neurons (MND) in the cortex, brainstem, and spinal cord [1]. ALS typically involves simultaneous upper (UMN) and lower motor neurons (LMN) in four different body regions (bulbar, cervical, thoracic, and lumbosacral) and is usually fatal within 4 years from disease onset [1]. The varying association of UMN and LMN signs makes the clinical presentation and individual survival times highly heterogeneous, ranging from a rapidly progressive disease (with a survival rate of less than three years) to a very slow progressive form in 10–20% of cases [1,2]. In this regard, several studies have investigated the effects of the combination of UMN and LMN burden on the clinical and prognostic features of ALS patients. UMN signs in ALS tend to follow either a vertical pattern, originating from the cervical or lumbar region and extending to the ipsilateral leg or arm, or a non-contiguous pattern. In contrast, LMN signs typically manifest in a contiguous and horizontal manner, spreading from one limb to the contralateral one. These distinct patterns of UMN and LMN involvement provide important clinical information regarding the localization and spread of motor neuron pathology [3,4].

Furthermore, the degree of impairment of UMN and LMN can determine the ALS phenotype ranging from classic ALS, in which both UMN and LMN signs are equally affected, to phenotypes characterized by a predominance of UMN or LMN signs [5]. These distinct phenotypes are associated with different clinical presentation and disease progression [6,7]. Bearing in mind these observations, a comprehensive assessment of UMN and LMN signs in the clinical examination of ALS patients is of the utmost importance, both for identifying individuals for clinical trials and for developing personalized interventions [3,4]. Over the past decades, several clinical scoring systems have been developed to assess UMN and LMN impairment in ALS. Among these, the most widely adopted are the scale proposed by Devine et al. [8] for LMN signs and the Penn Upper Motor Neuron Scale [9] for UMN signs. Unfortunately, the above-mentioned LMN scale considers bulbar LMN involvement solely as the presence/absence of signs without providing the degree of bulbar involvement [8]. Aiming to fill in this clinical and research gap, we developed and validated a novel scale for rating the severity of LMN signs in the bulbar region.

## 2. Materials and Methods

We enrolled 195 consecutive patients (89 subjects of the female gender, 45.647%; mean age at the first assessment was 62.5 ± 11.3 years) who were referred to a tertiary medical center in Southern Italy from January 2014 to January 2023, and who received a final diagnosis of clinically definite, probable, possible, or suspect ALS according to the El Escorial criteria [10]. All of the patients underwent a standardized clinical assessment, including the collection of demographic and clinical information (i.e., date and the type of onset, date of diagnosis, and disease duration) and an assessment of the disease severity using ALSFRS-R [11], King’s stage [12], and Milano–Torino Staging (MiToS) [13].

Routine blood analysis, transcranial magnetic stimulation, electromyography, 3T brain and spinal cord MRI, and a cerebrospinal fluid analysis were performed to rule out any ALS-mimicking disorders. Longitudinal clinical evaluations were conducted at 4–6 month intervals and data regarding tracheostomy, positioning of PEG, or death were recorded. The censorship date was set at 31 March 2023. Tracheostomy, positioning of PEG, or death (if occurred) were considered as outcomes. The study was approved by the Ethics Committee for Medical Research at Azienda Sanitaria Locale Lecce on 25 May 2017 (approval number 6) and was performed following the Helsinki declaration and its later amendments. All subjects provided written informed consent.

### 2.1. Scale Development

The El Escorial criteria (1994) defined bulbar LMN involvement as the presence of atrophy, weakness, and fasciculations over the bulbar structures, among which include the jaw, face, palate, tongue, and larynx [10]. Accordingly, we developed a scoring system to assess the LMN signs in the bulbar region, with a progressive degree of severity ranging from 0 (no involvement) to 3 (significant, severe involvement). The score has been assigned as follows: 0 (not clinically relevant), 1 (definite but trace severity, presence of atrophy and/or fasciculation), 2 (moderate severity, 1st score criteria plus presence of tongue/larynx hyposthenia/hypomobility), and 3 (significant/severe involvement, 2nd score criteria plus presence of jaw and/or face weakness) (for a detailed description, see Table 1). Ten raters, including neurologists specializing in ALS and in neurocognitive disorders, retrospectively applied the scale to the medical records of the first evaluation of 195 ALS patients.

### 2.2. Statistical Analysis

The data were explored using descriptive statistics (mean ± standard deviation). Differences between the male and female patients with ALS were assessed using the independent sample *t*-test and the chi-squared test, as appropriate. Cohen’s kappa (Cohen’s *k*) and an intraclass correlation coefficient (ICC) were used to assess the inter-rater reliability. Linear regression was used to assess the association between the scale score, the ALSFRS-R total score, and the ALSFRS-R bulbar subscore. Finally, Kaplan–Meier survival curves were used to test the survival distribution according to the bulbar scale scores. All statistical analyses were performed with IBM SPSS Statistics v.20. *p*-value < 0.05 was considered statistically significant.

## 3. Results

Demographic and clinical features of the study cohort, divided according to gender, are reported in Table 2. No significant differences emerged in age, disease duration, ALSFRS-R, and King’s stage distribution between males and females. Male subjects with ALS were more likely to have clinically possible ALS (*p* < 0.05) and spinal onset (*p* < 0.05) than female ALS subjects. The site of symptoms onset was in the bulbar region in 33.8% of cases (*n* = 66). Definite and probable ALS resulted in 59.5% (*n* = 116) of the entire cohort. The mean disease duration was 21.6 months and the overall ALSFRS-R score and ALSFRS-R bulbar subscore were 35.6 and 8.8, respectively. MiTos stage 4 was observed in 13.8% of patients (*n* = 27) and King’s stage 4 was observed in 31.3% of patients (*n* = 61).

**Figure 1:**
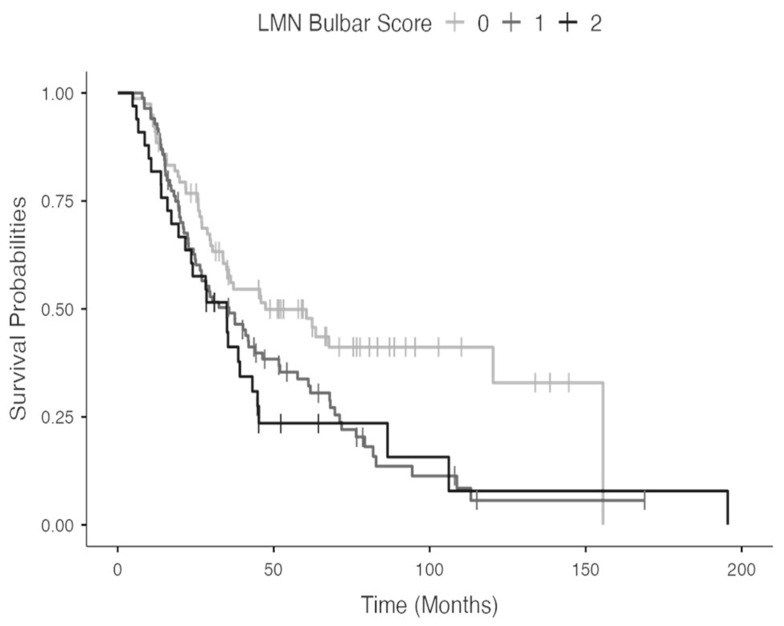
Survival distribution according to the bulbar scale score.

Cohen’s kappa coefficients for all raters are reported in Table 3. The raters showed substantial to excellent agreement with Cohen’s *k* ranging from 0.834 to 0.975, with an overall ICC of 0.922 (95% CI = 0.906–0.936). The linear regression analysis disclosed a significant association between the scale score and the ALSFRS-R bulbar subscore (R^2^: 0.207 *p* < 0.05), while the scale score did not result in a significant association with the ALSFRS-R total score (R^2^: 0.029 *p* = ns). The results of the log rank test assessing the differences in the survival distribution according to the bulbar scale score are shown in Figure 1. The survival distribution was statistically different across the three bulbar scale scores (χ^2^_(2)_ = 9.50, *p* < 0.01).

## 4. Discussion

In this study, we proposed a novel scale designed to assess the degree of LMN involvement in the bulbar region in ALS. Overall, the scale proved to be a reliable tool, as reflected by the substantial to excellent inter-rater agreement and the significant association with the ALSFRS-r bulbar subscore. Furthermore, the bulbar scale has been proven to be able to capture the overall survival associated with bulbar LMN involvement.

Involvement of the bulbar LMN in ALS is associated with a wide range of functional disabilities, including speech and swallowing dysfunctions, leading to dysphagia and aspiration pneumonia [14]. However, although the burden of the LMN and UMN involvement across the four body regions and the bulbar onset of symptoms are well-established factors influencing the prognosis and survival in ALS, the influence of LMN in the bulbar region on patients’ survival has yet to be elucidated.

The paucity of studies on this topic is likely due to the lack of a clinical scale to assess bulbar LMN impairment. Indeed, several clinical scoring systems have been developed to assess LMN impairment in ALS, yet the most widely adopted LMN scale (i.e., the Devine scale), in contrast to the upper and lower limbs, does not provide a grading of the degree of bulbar LMN impairment and assesses signs in the bulbar region solely as a presence or absence [8,15]. In addition, previous studies failed to achieve a consensus regarding clinical and paraclinical exams that are able to quantify the bulbar LMN involvement [16].

The simple four-items scale for the assessment of bulbar LMN burden in ALS that is proposed in this study proved to be a reliable tool as we observed an excellent agreement between the raters, regardless of their clinical experience with ALS. Furthermore, a higher burden of LMN bulbar signs, as assessed through the bulbar scale, resulted in an association with a lower ALSFRS-R bulbar subscore and a lower estimated survival.

These results suggest that the bulbar scale might present a suitable tool for bulbar LMN impairment assessment in clinical settings, as a complementary tool to the Devine scale. The scale proposed herein is indeed easy to administer in clinical practice as it does not require any instrumental assessment but relies only on clinical examination, and it is based on the LMN definition proposed in the El Escorial criteria [10] which are widely adopted in the clinical practice and in most clinical trials [17,18].

Furthermore, this tool may be useful to better identify patients with ALS which require therapeutic interventions (e.g., positioning of percutaneous endoscopic gastrostomy or tracheostomy) and to stratify subjects during clinical trials.

Some limitations of the study should be acknowledged. First, our center is a tertiary medical center and may be affected by selection bias, but it is worth noting that our center provides care for ALS patients to our entire region. Second, none of our subjects reached a score of 3 as the scale was applied only during the first evaluation. Third, we reported on a preliminary retrospective study and the clinical usefulness of the bulbar scale, which should be further validated in a prospective study. Finally, our sample size is relatively small, and although inclusive of different ALS phenotypes and stages, studies with a larger sample of patients are needed to further validate the bulbar scale.

## 5. Conclusions

In conclusion, the bulbar scale proposed here may represent a useful tool to assess LMN impairment in the bulbar region in ALS. A systematic quantitative assessment of bulbar LMN impairment may provide useful data about its prognosis. Indeed, the three bulbar scale scores showed significant differences among the survival distribution. Moreover, our scale is consistent with the natural progression of ALS, further confirming that the LMN burden is closely related to the global disease burden and reliably predicts the long-term survival. 

## Figures and Tables

**Table 1:** Score assessment of LMN signs in bulbar region.

LMN Signs	Score
**Not clinically significant**	0
**Tongue atrophy and/or fasciculations**	1
**Score (1) + tongue hypomobility**	2
**Score (2) + jaw and/or face weakness**	3

LMN: lower motor neuron.

**Table 2:** Clinical and demographic characteristics of ALS population.

	Male ALS Patients (*n* = 106)	Female ALS Patients (*n* = 89)	*p* Value	
	Mean ± SD	Mean ± SD		
Age, *y*	62.7 ± 11.5	62.5 ± 11.3	ns	
Disease duration, months	22.2 ± 22	21 ± 18.6	ns	
Diagnostic category *				
Clinically definite ALS	35 (33%)	34 (38.2%)	<0.05	
Clinically probable ALS	19 (17.9%)	28 (31.5%)		
Clinically possible ALS	41 (38.7%)	20 (22.5%)		
Clinically suspect ALS	11 (10.4%)	7 (7.9%)		
Type of onset				
Bulbar	29 (27.4%)	37 (41.6%)	<0.05	
Spinal	77 (72.6%)	52 (58.4%)		
ALSFRS-R	35.9 ± 9	35.3 ± 7.6	ns	
ALSFRS-R Bulbar subscore	9 ± 3.1	8.7 ± 3.2	ns	
King’s staging				
Stage 0	1 (0.9%)	2 (2.2%)	ns	
Stage 1	12 (11.3%)	8 (9%)		
Stage 2	27 (25.5%)	14 (15.7%)		
Stage 3	35 (33%)	35 (39.3%)		
Stage 4	31 (29.2%)	30 (33.7)		
Milano–Torino staging (MiToS)				
Stage 0	23 (21.7%)	5 (5.6%)	<0.05	
Stage 1	37 (34.9%)	48 (53.9%)		
Stage 2	11 (10.4%)	16 (18%)		
Stage 3	17 (16%)	11 (12.4%)		
Stage 4	18 (17%)	9 (10.1%)		

* According to the El Escorial criteria of 1994; ALS: amyotrophic lateral sclerosis; ALSFRS-R: Revised Amyotrophic Lateral Sclerosis Functional Rating Scale; ns: not significant.

**Table 3:** Cohen’s kappa coefficients between all raters.

	Rater 1	Rater 2	Rater 3	Rater 4	Rater 5	Rater 6	Rater 7	Rater 8	Rater 9	Rater 10
Rater 1	-	0.943	0.959	0.967	0.902	0.975	0.853	0.894	0.901	0.859
Rater 2	0.943	-	0.918	0.934	0.910	0.935	0.878	0.935	0.926	0.868
Rater 3	0.959	0.918	-	0.942	0.910	0.934	0.861	0.869	0.892	0.850
Rater 4	0.967	0.934	0.942	-	0.894	0.942	0.845	0.885	0.892	0.850
Rater 5	0.902	0.910	0.910	0.894	-	0.894	0.919	0.878	0.885	0.860
Rater 6	0.975	0.935	0.934	0.942	0.894	-	0.861	0.886	0.893	0.834
Rater 7	0.853	0.878	0.861	0.845	0.919	0.861	-	0.845	0.868	0.844
Rater 8	0.894	0.935	0.869	0.885	0.878	0.886	0.845	-	0.893	0.835
Rater 9	0.901	0.926	0.892	0.892	0.885	0.893	0.868	0.893	-	0.866
Rater 10	0.859	0.868	0.850	0.850	0.860	0.834	0.844	0.835	0.866	-

## Data Availability

The data that support the findings of this study are available from the corresponding author upon reasonable request.
